# Alcohol drinking patterns and liver cirrhosis risk: analysis of the prospective UK Million Women Study

**DOI:** 10.1016/S2468-2667(18)30230-5

**Published:** 2018-11-22

**Authors:** Rachel F Simpson, Carol Hermon, Bette Liu, Jane Green, Gillian K Reeves, Valerie Beral, Sarah Floud

**Affiliations:** a Cancer Epidemiology Unit, Nuffield Department of Population Health, University of Oxford, UK; b School of Public Health and Community Medicine, University of New South Wales, Sydney 2052, Australia

## Abstract

**Background:**

Alcohol is a known cause of cirrhosis, but it is unclear if the associated risk varies by whether alcohol is drunk with meals, or by the frequency or type of alcohol consumed. Here we aim to investigate the associations between alcohol consumption with meals, daily frequency of consumption, and liver cirrhosis.

**Methods:**

The Million Women Study is a prospective study that includes one in every four UK women born between 1935 and 1950, recruited between 1996 and 2001. In 2001 (IQR 2000–03), the participants reported their alcohol intake, whether consumption was usually with meals, and number of days per week it was consumed. Cox regression analysis yielded adjusted relative risks (RRs) for incident cirrhosis, identified by follow-up through electronic linkage to routinely collected national hospital admission, and death databases.

**Findings:**

During a mean of 15 years (SD 3) of follow-up of 401 806 women with a mean age of 60 years (SD 5), without previous cirrhosis or hepatitis, and who reported drinking at least one alcoholic drink per week, 1560 had a hospital admission with cirrhosis (n=1518) or died from the disease (n=42). Cirrhosis incidence increased with amount of alcohol consumed (≥15 drinks [mean 220 g of alcohol] *vs* one to two drinks [mean 30 g of alcohol] per week; RR 3·43, 95% CI 2·87–4·10; p<0·0001). About half of the participants (203 564 of 401 806) reported usually drinking with meals and, after adjusting for amount consumed, cirrhosis incidence was lower for usually drinking with meals than not (RR 0·69, 0·62–0·77; p<0·0001; wine-only drinkers RR 0·69, 0·56–0·85; all other drinkers RR 0·72, 0·63–0·82). Among 175 618 women who consumed seven or more drinks per week, cirrhosis incidence was greater for daily consumption than non-daily consumption (adjusted RR 1·61, 1·40–1·85; p<0·0001). Daily consumption, together with not drinking with meals, was associated with more than a doubling of cirrhosis incidence (adjusted RR 2·47, 1·96–3·11; p<0·0001).

**Interpretation:**

In middle-aged women, cirrhosis incidence increases with total alcohol intake, even at moderate levels of consumption. For a given weekly intake of alcohol, this excess incidence of cirrhosis is higher if consumption is usually without meals, or with daily drinking.

**Funding:**

UK Medical Research Council and Cancer Research UK.

## Introduction

Alcohol is a known cause of liver cirrhosis, with its incidence increasing in relation to the total amount of alcohol consumed.^1^, ^2^, ^3^, ^4^, ^5^, ^6^, ^7^ Evidence for how cirrhosis risk is affected by drinking habits, such as whether or not alcohol is usually drunk with meals or is consumed every day or less often, or by the type of alcohol consumed, is scarce.^7^, ^8^, ^9^, ^10^, ^11^, ^12^ For example, we found only one report that had examined associations with drinking with meals—a cross-sectional study of 35 cases of cirrhosis or hepatocellular cancer.^8^ Drinking with meals and the frequency of consumption are correlated with the total intake of alcohol, so large numbers of study participants are needed to examine the separate effects of different drinking practices reliably, allowing for the effects of the correlated exposures.

The Million Women Study includes one in every four UK women born between 1935 and 1950.^13^ In this prospective study, the weekly amount of alcohol consumed has been shown to be a strong predictor of cirrhosis incidence, even at the moderate levels of alcohol intake that are typical for UK women of this generation.^6^ Information on other drinking habits was also collected in this study. Our aim was to investigate associations between liver cirrhosis risk and alcohol consumption with meals, the number of days per week alcohol was consumed, and the type of beverage drunk, taking careful account of total alcohol intake, other related drinking habits and possible confounders.

## Methods

### Study design and participants

The Million Women Study is a prospective study that includes 1·3 million UK women, mostly between age 50 and 64 years when they were recruited from May 1, 1996, to Dec 31, 2001, through the UK National Health Service (NHS) Breast Screening Programme by completing an initial study questionnaire.^13^ The women have been sent postal re-survey questionnaires every 3–5 years thereafter. Every questionnaire asks about alcohol consumption. Questions about drinking with meals and the number of days per week alcohol was consumed were asked for the first time about 3 years after recruitment, in median year 2001 (IQR 2000–03), and this 3-year re-survey was the baseline for these analyses. The questionnaires and data access policies can be viewed on the study website.^14^ From 2010 onwards, women who provided a valid email address were also asked to complete a 24-h recall of diet, including alcohol consumption, on a randomly selected day of the week.^15^

**Research in context**

**Evidence before this study**

We searched MEDLINE with a mixture of MeSH subject headings and keywords (“alcohol”, “alcohols”, “ethanol”, “alcohol consumption”, “alcoholic beverages”, or “alcohol drinking”) and (“hepatic cirrhosis” or “liver cirrhosis”) for meta-analyses or prospective studies with more than 100 cases of cirrhosis in humans from 1946 to Jan 20, 2018. Because of the small number of prospective studies with relevant results on mealtime habits and frequency of consumption, we expanded the search to any study if relative risks or the equivalent were given. Further searches were done by reading papers identified as suitable from database and other searches. Many prospective studies and two meta-analyses from 2004 and 2010 reported increasing risks of cirrhosis with increasing levels of alcohol consumption. However, there was little published information on associations with mealtime habits or the frequency of consumption.

**Added value of this study**

This large prospective study with long follow-up permitted detailed examination of cirrhosis risk in relation to the amount of alcohol consumed, consumption with meals, and the frequency of consumption, adjusted for drinking patterns and other potential confounding factors. During a mean of 15 years (SD 3) of follow-up of 401 806 UK women who consumed alcohol, 1560 developed or died from cirrhosis. As expected, cirrhosis risk increased with the amount of alcohol consumed. After allowing for the amount consumed, the excess risk of cirrhosis was consistently lower among those who usually drank alcohol with meals, both overall (RR 0·69, p<0·0001) and separately in women who drank wine only and all other drinkers. Among women who drank seven or more alcoholic drinks per week, the excess risk of cirrhosis was greater with daily consumption than with less frequent consumption (RR 1·61, p<0·0001). Drinking daily and not with meals was associated with a doubling of cirrhosis risk.

**Implications of all the available evidence**

Our study confirms that even moderate levels of alcohol consumption increase the risk of cirrhosis. It also shows that, after adjusting for the amount of alcohol consumed, this excess incidence of cirrhosis is higher if consumption is usually without meals or with daily drinking.

Women were excluded from the analyses if, before baseline, they were registered with cancer, except non-melanoma skin cancer (n=47 378), or if, at or before baseline, they self-reported or had a hospital admission with cirrhosis or chronic hepatitis (WHO International Classification of Diseases tenth revision [ICD-10] codes K70, K73, and K74; n=1369) or viral hepatitis (ICD-10 B15–B19; n=723).^16^ Women were also excluded if, at baseline, they did not report the number of alcoholic drinks they consumed per week (n=106 329) or if they did not report whether they usually drank the alcohol with meals (n=42 114).

The study was approved by the Oxford and Anglia multicentre research ethics committee, and all participants provided written consent for follow-up. Access to hospital admission data was approved by NHS Digital in England and by the Information Services Division in Scotland.

### Procedures

Women were asked the number of alcoholic drinks they consumed per week; if they usually drank alcohol with meals, without meals, or if it varied; and the number of days per week on which they usually drank alcohol. Participants were requested to report zero if they drank less than one alcoholic drink per week and they are defined here as non-drinkers. Drinkers are defined as those who reported drinking one or more alcoholic drinks per week. One drink was specified in the questionnaire as one glass of wine, half a pint of lager, or a tot of spirits. Separate questions about the consumption of wine, spirits, and lager, beer, or cider were asked at recruitment but not repeated 3 years later.

Drinkers were grouped into four categories of reported alcohol consumption (drinks per week) at baseline: one to two, three to six, seven to 14, and 15 or more. To allow for changes in alcohol consumption over time, measurement error and regression dilution bias, repeat measures of alcohol intake were used to derive mean intakes in g/week within each of the four baseline groups (appendix p 4).^17^ The repeat measures were taken from alcohol intake reported online in a 24-h recall of diet, which included questions about alcohol intake, sent to participants on randomly selected days of the week; responses were completed for the day specified by 19 293 analysis participants, in 2010–17, a mean of 11 (SD 2) years after they completed the baseline questionnaire (appendix p 4).

The analyses focused on women who reported drinking at least one alcoholic drink per week, because the majority of women reporting drinking less than one drink per week were likely to be ex-drinkers (in a subsequent questionnaire, completed 9 years after baseline for these analyses, only about one out of every seven who reported drinking no drinks per week at baseline were lifelong non-drinkers). Ex-drinkers might have stopped drinking because of poor health and could differ from drinkers in ways that might be hard to measure but are potentially linked to the development of cirrhosis.^18^

Using each individual's unique NHS number, participants were followed by electronic record linkage to routinely collected NHS data on deaths, emigrations, and hospital admissions (by NHS Digital in England^19^ and by the Information Services Division in Scotland^20^). Diagnoses were coded to ICD-10.^16^

### Outcomes

Participants were classified as having liver cirrhosis (ICD-10 K70 or K74) if they had either a hospital admission where cirrhosis was recorded or if the disease was listed as the underlying cause of death.

### Statistical analysis

For the 401 806 women included in this analysis, person-years were calculated from the date that the baseline questionnaire was completed to the earliest of the following: hospital admission with cirrhosis, emigration, death, or end of follow-up, which was March 31, 2017 (the last date when follow-up was complete).

Cox proportional hazard models were used, with time since baseline as the underlying time variable, to estimate the hazard ratios (referred to as relative risks [RRs]) and their 95% CIs for cirrhosis. When more than two groups were compared, group-specific CIs were calculated to allow direct comparison between any two groups.^21^ Conventional CIs are quoted in the text.

To ensure that comparisons were made within women who were as similar as possible, all analyses were stratified by single year of birth (1930 or earlier, individual years from 1931 to 1949, 1950 or later) and single year of completing the baseline questionnaire (2000 or earlier, 2001, 2002, 2003, 2004 or later), and were adjusted for five regions of residence in the UK (London and Southeast, Southwest, Midlands, Northern England, Scotland), deprivation quintile (according to the Townsend index^22^), smoking (never, past, or current; if current, <ten, ten to 19, ≥20 cigarettes per day), oral contraceptive use (ever or never), hormone replacement therapy use (never, past, current, or ever), and body-mass index (BMI; <22·5, 22·5–24·9, 25·0–27·4, 27·5–29·9, or ≥30 kg/m^2^). Adjustment variables were from baseline except for deprivation, oral contraceptive use, and height for BMI, which were from recruitment. So that the same women were included in all analyses, the small numbers with missing data for each adjustment variable were included as a separate category. Where appropriate, analyses were also adjusted for the number of alcoholic drinks consumed per week reported at baseline (one to two, three to six, seven to 14, and ≥15), type of alcohol usually consumed (wine-only drinkers or all other drinkers), mealtime habits (usually consume alcohol with meals; do not usually consume alcohol with meals, or it varies), and frequency of consumption (daily or non-daily).

For analyses of the amount of alcohol consumed, those who reported drinking one to two alcoholic drinks per week were the reference category. For analyses of RR associations with mealtime habits, the reference category was women who reported drinking without meals. For analyses of associations with the frequency of consumption, those consuming fewer than seven drinks per week were excluded (as their typical daily intake would, by definition, be less than one drink a day) and the reference category was women who reported non-daily consumption. For comparisons of the combined effect of frequency and mealtime habits, the reference category was non-daily consumption of alcohol, usually with meals.

We did sensitivity analyses for the effect of mealtime habits and frequency of consumption, including women with missing values for alcohol intake at baseline by substituting reported alcohol intake at recruitment. We also assessed these associations separately in subgroups defined by women's BMI (<25 kg/m^2^ *vs* ≥ 25 kg/m^2^) and smoking status (current smokers *vs* never smokers).

Stata, version 15.1, was used for all analyses. All statistical tests were two-sided. Statistical significance was defined at a p value of less than 0·05.

### Role of the funding source

This work was funded by the UK Medical Research Council and Cancer Research UK. Funders had no role in the design of the study, data collection, data analysis, data interpretation, writing of the report, or the decision to publish. RFS and CH had access to all data in the study and all authors gave approval to submit for publication.

## Results

**Figure 1:**
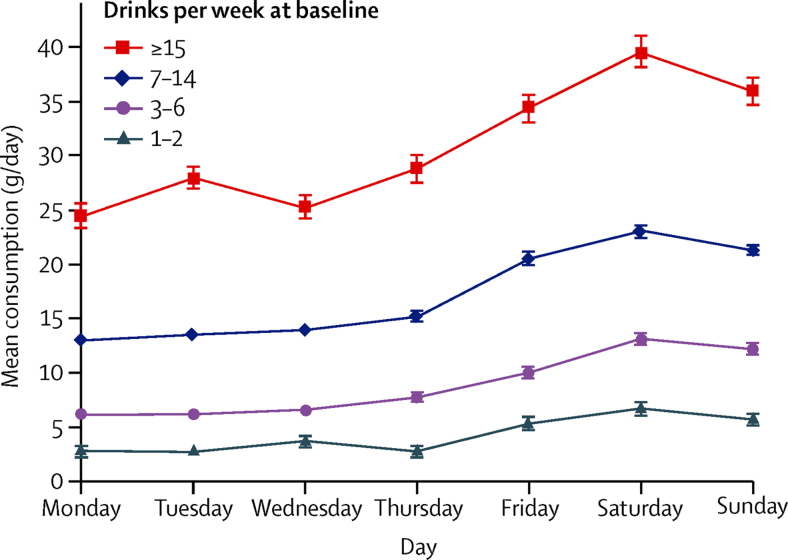
Mean alcohol consumption for 24-h recall of alcohol intake on randomly selected days of the week 11 years after baseline, by category of consumption per week reported at baseline Bars indicate standard errors.

Among the 401 806 women without previous liver disease who reported consuming at least one alcoholic drink per week, 71 649 (18%) reported consuming one to two drinks per week, 149 523 (37%) three to six drinks per week, 142 762 (36%) seven to 14 drinks per week, and 37 872 (9%) 15 or more drinks per week. For each of these four baseline categories we calculated the mean daily alcohol intake (g/day) based on 24-h recall of alcohol intake on randomly selected days of the week, reported 11 years after baseline (figure 1). For every baseline consumption category, the average daily intake was greater on Fridays, Saturdays, and Sundays than on other days. For every baseline category, weekly intakes assessed 11 years after baseline agreed with the weekly baseline intakes, with slight regression to the mean: for one to two, three to six, seven to 14, and 15 or more drinks per week, the mean remeasured values were 2·5, 5·2, 10·0, and 18·0 drinks per week, respectively; ie, 30, 62, 120, and 216 g/week. Information on the type of alcohol consumed at recruitment also agreed with that reported 11 years after baseline; for example, among wine-only drinkers at recruitment who reported at 24-h recall that they had drunk alcohol the previous day, 3480 (78%) of 4486 reported drinking wine and no other alcohol type that day.

**Figure 2:**
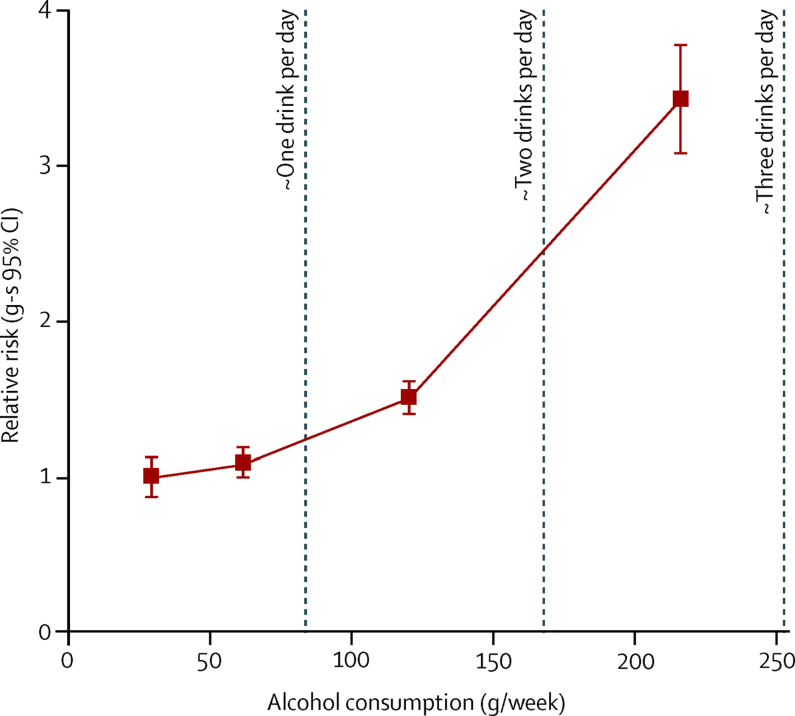
Relative risk (RR) of liver cirrhosis by the amount of alcohol consumed RR and group-specific (g-s) 95% CIs for liver cirrhosis by amount of alcohol consumed compared with consumption of one to two drinks (mean 31 g) per week (RR 1·0), adjusted for region, body-mass index, deprivation quintile, smoking, use of oral contraceptives and menopausal hormones, and stratified by year of birth and year completed baseline questionnaire. The RRs are for categories of one or two, three to six, seven to 14, 15 or more drinks per week plotted against the remeasured averages in each category (30, 62, 120, and 216 g/week respectively).

During 5 835 149 person-years of follow-up, a mean of 15 (SD 3) years per woman, 1560 had a first record of cirrhosis at a hospital admission (n=1518) or as the underlying cause of death (n=42). Cirrhosis risk increased with the total amount of alcohol consumed per week (figure 2). The adjusted RR of cirrhosis for 15 or more versus one to two drinks per week (ie, for an average of 220 g *vs* 30 g of alcohol per week) was 3·43 (95% CI 2·87–4·10; p<0·0001).

Overall, about half of the women (203 564 [51%] of 401 806) reported usually drinking with meals and the remaining half reported not usually drinking with meals, or in a varied manner. Women who usually drank with meals consumed slightly less alcohol (83 g/week) than women who did not usually drink with meals (95 g/week) at baseline; they were also less likely to be current smokers and live in deprived areas (table). These factors are, however, considered in analyses of mealtime drinking and cirrhosis risk. The intakes within each baseline category reported at 24-h recall were similar for those who, at baseline, reported usually drinking with meals and for those who reported drinking without meals (appendix p 6).

**Table:** Alcohol intake and other characteristics of women included in the analyses, by reported consumption of alcohol with meals and frequency of consumption

		**Alcohol consumption with meals**		**Frequency of alcohol consumption (restricted to women reporting seven or more drinks per week)**	
		Usually with meals (n=203 564)	Not usually with meals or varies (n=198 242)	Less than daily (n=96 863)	Daily (n=78 755)
**Alcohol consumption**					
Baseline*					
	All women, g/week	83 (63)	95 (76)	134 (52)	161 (81)
	Drinkers of wine only†, g/week	78 (61)	94 (77)	134 (51)	154 (75)
	Other drinkers†, g/week	87 (64)	97 (76)	134 (53)	165 (84)
11 years after baseline‡					
	All women, g/week	98 (44)	107 (50)	130 (49)	162 (54)
**Characteristics**					
Baseline					
	Age, years	60 (5)	59 (5)	59 (4)	60 (5)
	Most deprived quintile†	17 978 (9%)	31 676 (16%)	12 535 (13%)	7627 (10%)
	Body-mass index, kg/m^2^	25 (4)	26 (4)	25 (4)	25 (4)
	Current smoker	13 440 (7%)	29 018 (15%)	11 860 (12%)	10799 (14%)
	Current users of menopausal hormones	61 471 (31%)	58 667 (30%)	30 616 (32%)	24 977 (32%)
Follow-up					
	Person-years	2 962 464	2 872 685	1 405 790	1 132 223
	Years of follow-up per woman	14·6	14·5	14·5	14·4
	Incident cases of cirrhosis	547	1013	381	519

Data are mean (SD) or n (%), unless otherwise specified. Percentages exclude the small number with missing values: age (0%); body-mass index (n=24 199, 6%); smoking (n=5073, 1%); menopausal hormone use (n=6796, 2%); deprivation (n=3062, 1%); alcohol type (n=7906, 2%).

* One drink at baseline was defined as one glass of wine, half a pint of lager, or a tot of spirits and assumed to equal 12 g of alcohol. All values quoted are at baseline (3·3 years after recruitment), unless otherwise indicated.

† From information collected at recruitment.

‡ From information collected by 24-h recall 11 years after baseline.

**Figure 3:**
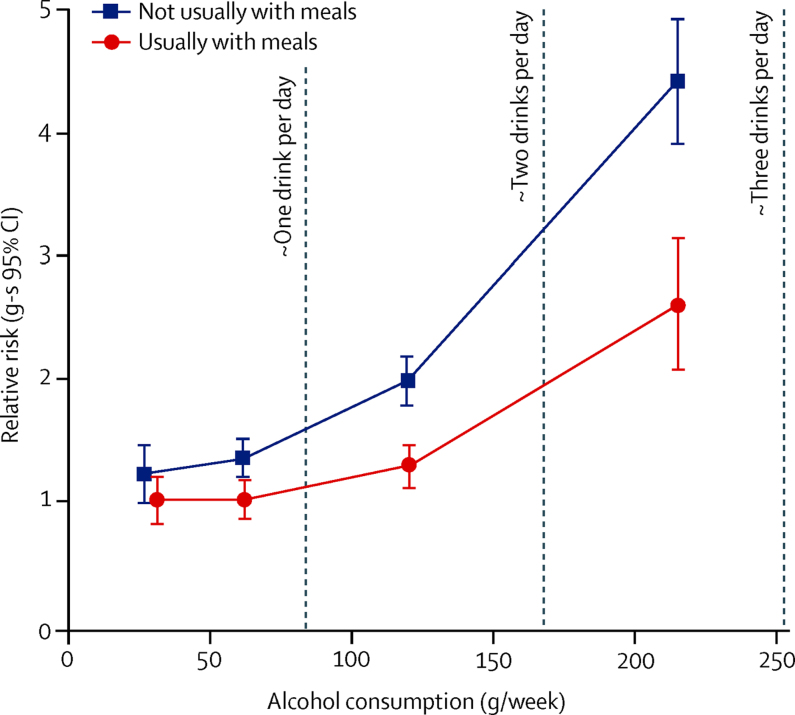
Relative risk (RR) of liver cirrhosis by the amount of alcohol consumed and whether it was usually with meals RR and group-specific (g-s) 95% Cls for liver cirrhosis by amount of alcohol consumed compared with consumption of one to two drinks (mean 31 g) per week with meals (RR 1·0), adjusted for region, body-mass index, deprivation quintile, smoking, use of oral contraceptives and menopausal hormones, and stratified by year of birth and year completed baseline questionnaire. The RRs are for categories of one or two, three to six, seven to 14, and 15 or more drinks per week plotted against the remeasured averages in each category (with meals 31, 62, 121, and 216 g/week, respectively; without meals 27, 61, 120, and 215 g/week, respectively).

**Figure 4:**
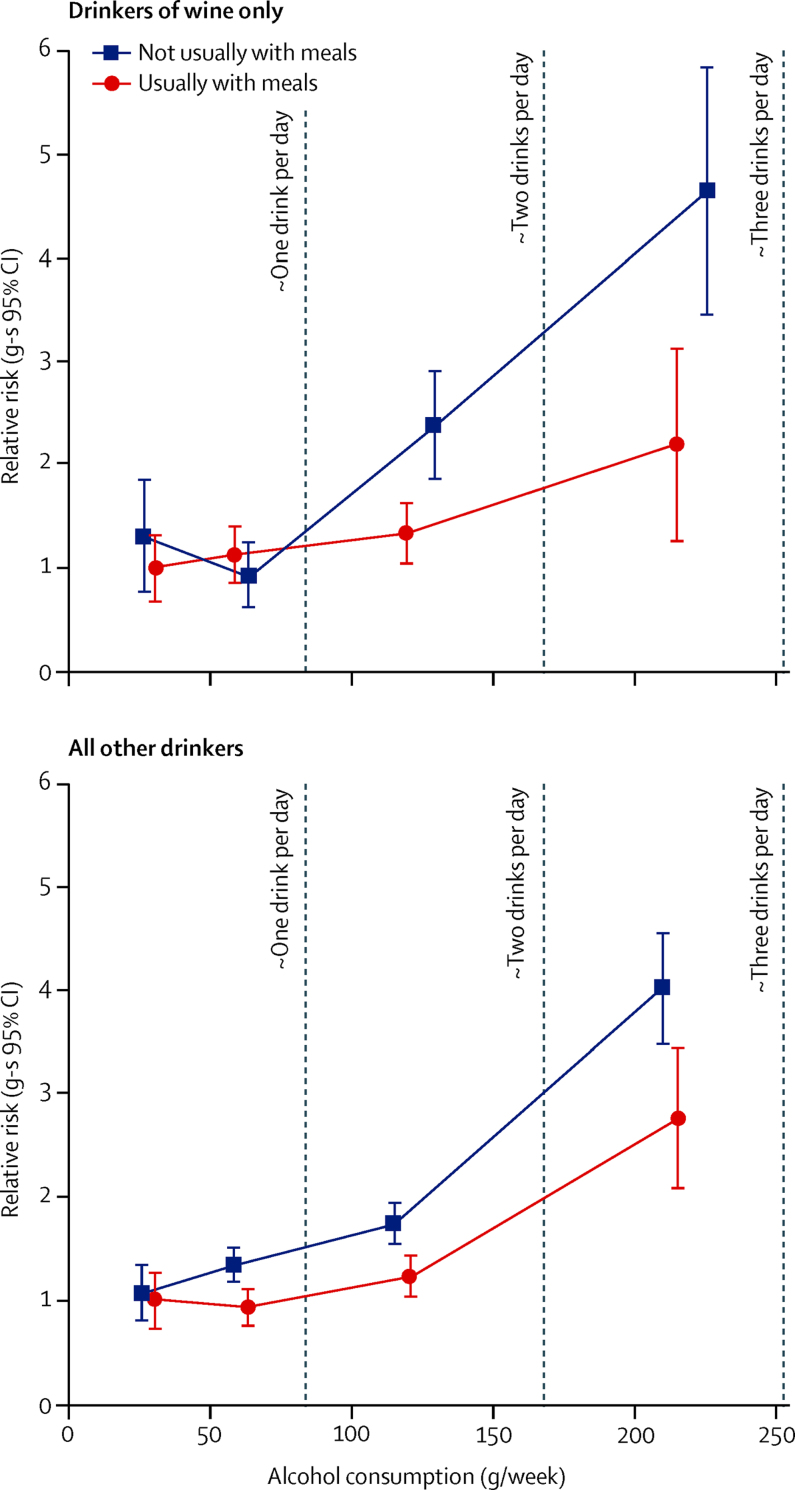
Relative risk (RR) of liver cirrhosis by amount of alcohol consumed, whether it was usually with meals, and the type of alcohol RR and group-specific (g-s) 95% Cl for liver cirrhosis by amount of alcohol consumed compared with consumption of one to two drinks (mean 31 g) per week with meals (RR 1·0) for wine drinkers exclusively and all other drinkers separately, adjusted for region, body-mass index, deprivation quintile, smoking, use of oral contraceptives and menopausal hormones, and stratified by year of birth and year completed baseline questionnaire. The RRs are for categories of one or two, three to six, seven to 14, and 15 or more drinks per week plotted against the remeasured averages in each category (wine only: with meals 31, 59, 120, and 214 g/week, respectively, without meals 27, 64, 129, and 226 g/week, respectively; all other drinkers: with meals 32, 65, 122, and 216 g/week, respectively, and without meals 28, 60, 117, and 211 g/week, respectively).

At every level of alcohol consumption, cirrhosis incidence was lower in women who usually drank with meals compared with those who did not (figure 3). After adjusting for the amount of alcohol consumed (one to two, three to six, seven to 14, and ≥15 drinks per week) and the six other potential confounding factors (ie, region, deprivation quintile, smoking, BMI, past oral contraceptive use, and use of menopausal hormones), the RR for cirrhosis associated with usually drinking with meals compared with not drinking with meals was 0·69 (95% CI 0·62–0·77; p<0·0001). Type of alcohol consumed had been reported by 393 900 (98%) of 401 806 women included in the analyses. Consumption of wine and no other alcohol type was more common in women who drank with meals (87 360 [44%] of 200 246) than in those who did not (48 268 [25%] of 193 654). Nevertheless, both among wine-only drinkers and also among all other drinkers, cirrhosis risk was lower in women who usually drank with meals than in women who did not (figure 4). After adjustment for the amount of alcohol consumed and the other factors, the RRs associated with drinking with meals were similar in drinkers of wine only (RR 0·69, 95% CI 0·56–0·85) and in all other drinkers (RR 0·72, 0·63–0·82); RRs associated with meals also did not differ significantly by women's adiposity (RR 0·68, 0·57–0·81 for BMI of <25 kg/m^2^ and 0·68, 0·59–0·79 for BMI ≥25 kg/m^2^) or between current and never smokers (RR 0·84, 0·66–1·06 and 0·67, 0·56–0·80). Examination of the separate effect of every adjustment factor indicated that the main confounding was by the total amount of alcohol consumed and by cigarette smoking (appendix p 7).

**Figure 5:**
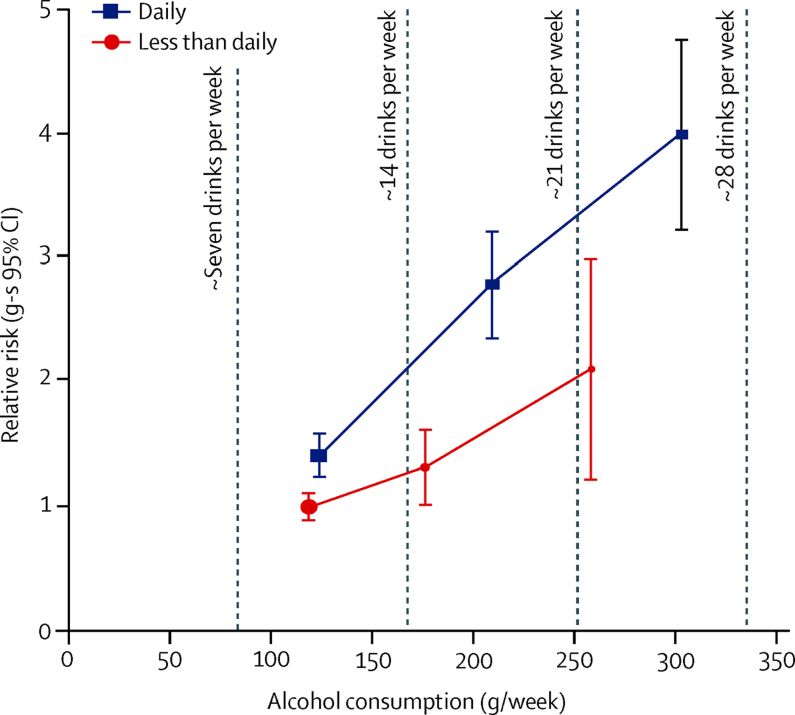
Relative risk (RR) of liver cirrhosis by amount of alcohol consumed and frequency of alcohol consumption, in women who consumed at least seven drinks per week RR and group-specific (g-s) 95% Cl for liver cirrhosis by amount of alcohol consumed compared with consumption of seven to 14 drinks (mean 119 g) per week less often than daily (RR 1·0), adjusted for region, body-mass index, deprivation quintile, smoking, use of oral contraceptives, menopausal hormones, meal time habits, and type of alcohol, and stratified by year of birth and year completed baseline questionnaire. The RRs are for categories of seven to 14, 15 to 21, and 22 or more drinks per week plotted against the remeasured averages in each category (less often than daily 119, 177, and 259 g/week, respectively; daily 124, 209, and 303 g/week, respectively).

Among the 175 618 women who reported drinking at least seven drinks per week and also reported frequency of consumption, those who drank daily had greater total intakes of alcohol (161 g/week) than non-daily drinkers (134 g/week; table). There was little difference in other characteristics between the two groups. For a given alcohol intake, the RR of cirrhosis was greater in those who reported drinking alcohol daily than those who drank less often than daily (figure 5). After adjustment for the total amount consumed (seven to 14, 15–21, and ≥22 drinks per week), mealtime habits, and type of alcohol, the RR for cirrhosis for daily versus less frequent consumption was 1·61 (95% CI 1·40–1·85; p<0·0001). Examination of the separate effect of each adjustment factor indicated that the greatest confounding was by the total amount of alcohol drunk and if consumption was with meals (appendix p 8). RRs did not differ significantly by women's adiposity (RR 1·51, 1·21–1·89 for BMI of <25 kg/m^2^ and 1·67, 1·37–2·03 for ≥25 kg/m^2^) or between current (RR 1·52, 1·18–1·97) and never smokers (RR 1·54, 1·18–2·01). Among women consuming at least seven drinks per week, the adjusted RR associated with drinking on 4–6 days versus fewer than 4 days per week was 1·27 (95% CI 1·01–1·59; p=0·041).

When mealtime habits and frequency of consumption were considered simultaneously, the RR of cirrhosis associated with drinking daily and not with meals more than doubled, after additional adjustment for type and amount of alcohol consumed (RR 2·47, 95% CI 1·96–3·11; p<0·0001).

In sensitivity analyses using reported alcohol at recruitment as a substitute for missing values of alcohol intake at baseline, the RRs for cirrhosis by mealtime habits or frequency of consumption were similar to the main results reported here (appendix pp 10, 11).

Among the 393 900 women for whom the types of alcohol consumed were recorded, most (225 766 [57%] of 393 900) reported consuming more than one type of alcohol; 135 628 (34%) of 393 900 reported drinking wine only, and relatively few reported consuming spirits only (22 020 [6%] 393 900) or lager, beer, or cider only (10 486 [3%] of 393 900; appendix p 9). The risk of cirrhosis for women drinking more than one type of alcohol compared with women drinking wine only was similar (RR 1·17, 1·03–1·32; p=0·013). The personal characteristics and drinking habits of the spirit-only and lager, beer, or cider-only drinkers differed substantially from those of the wine-only drinkers, in that only one in every seven drank with meals compared with two in every three wine-drinkers and they were about three to four times more likely to be smokers and live in deprived areas. This limits reliable assessment of cirrhosis risk in the small subgroups who reported consuming just spirits or just lager, beer, or cider, since considerable residual confounding by measured or unmeasured factors, or both, is likely.

## Discussion

In this large prospective study of UK women aged in their 50s and 60s at baseline, we found that the incidence of cirrhosis increased with the amount of alcohol consumed, even at moderate levels of consumption typical for women of this age.^23^ However, for a given amount of alcohol consumed, the excess risk of cirrhosis was lower, by about a third, if alcohol was usually consumed with meals than without meals (RR 0·69). In addition, among those consuming seven or more drinks per week, after adjusting for the amount drunk, whether or not it was with meals and type of alcohol consumed, the excess risk of cirrhosis was about two-thirds higher with daily than with less frequent consumption (RR 1·61). For a given amount of alcohol consumed, daily consumption together with not usually drinking with meals was associated with more than a doubling of cirrhosis incidence.

The large size of our study and its prospective design, along with the in-depth examination of associations with different patterns of alcohol consumption, offer insights into the effect of drinking habits that previous studies have been unable to provide. Mealtime drinking habits and the frequency of consumption are correlated with the total amount of alcohol consumed. With the large numbers of cases in this study, it was possible to show clearly that the lower excess risks associated with consumption with meals and non-daily alcohol intake were evident at every level of total consumption.

To ensure that similar women were compared, our analyses were routinely stratified by exact year of birth and calendar year when alcohol intake was reported, and were also adjusted for six potential confounding factors (ie, region of residence, deprivation, smoking, BMI, past oral contraceptive use, and use of menopausal hormones). Although different methods were used to assess total alcohol intake at baseline and 11 years later, the mean intakes in the baseline categories were similar to the means 11 years later, with slight regression to the mean. Assigning the reassessed mean value to each baseline category allowed for changes in drinking habits over time, measurement error, and regression dilution biases.^17^ For each pattern of alcohol consumption examined, results were also adjusted for other drinking habits. Confounding by the other drinking habits was generally found to be greater than confounding by the six other adjustment factors.

To our knowledge, this is the first prospective study to report on the association between mealtime alcohol consumption and incident liver cirrhosis. Our results, based on 1560 incident cases among drinkers, appear to concur with findings from a small cross-sectional study^8^ of just 35 cases of cirrhosis or hepatocellular cancer, which reported an increased risk for the two conditions combined in those not drinking with meals.

Regarding frequency of alcohol consumption, only two other prospective studies, one including 622 cases of cirrhosis,^7^ and the other including 285 cirrhosis deaths in alcohol misusers,^9^ provided estimates of risk and both suggested a possible increased risk of cirrhosis associated with daily consumption of alcohol in men. However, risk estimates were not adjusted for drinking with meals, which was an important confounding factor in our analysis.

More than half the women reported drinking more than one type of alcohol and a third reported drinking wine only and there was little difference in their risk of cirrhosis. The small proportions who reported drinking spirits only, or lager, beer, or cider only, differed substantially from the wine-only drinkers and drinkers of a mixture of alcoholic beverages both in terms of their personal characteristics and their drinking habits, which limits comparison of cirrhosis risk, because residual confounding by measured or unmeasured factors, or both, is possible. Findings from other studies on associations with the type of alcohol consumed are varied and inconsistent.^7^, ^8^, ^9^, ^10^, ^11^, ^12^

The mechanisms that underlie the associations observed here are unclear. With respect to the effect of meals, it has been suggested that delayed gastric emptying occurs in the presence of food, and that alcohol is absorbed more slowly in the intestine, leading to lower blood alcohol concentrations.^24^ For non-daily consumption, one possible explanation is that the break from alcohol consumption allows the liver time to recover after each episode of drinking.

Regarding limitations, although we were able to adjust each aspect of alcohol consumption by weekly alcohol consumption and other drinking habits, as well as by other potential confounding factors, some residual confounding cannot be excluded. We did not study men and did not have information about drinking habits at younger ages.^25^ Other limitations are that this cohort did not include large numbers of heavy drinkers, so we could not assess the effect of different patterns of irregular heavy drinking.

Strengths of this cohort, such as its large size and completeness of follow-up, have been detailed previously.^6^ The prospective design of the study should largely eliminate differential recall of alcohol intake between those who developed and did not develop cirrhosis during follow-up. Applying levels of alcohol consumption reassessed 11 years after baseline helped to minimise effects of changes in alcohol consumption over time, measurement error and regression dilution bias (figure 1).^17^ Analyses were restricted to drinkers who reported consuming at least one alcoholic drink per week at baseline. Only one in every seven non-drinkers at baseline were lifelong non-drinkers, and the rest were ex-drinkers who could have stopped drinking because of poor health, but for whom we do not have information on when or why they stopped; hence, excluding non-drinkers at baseline from the analyses would minimise reverse causation biases.

In conclusion, liver cirrhosis risk increases with amount of alcohol drunk, but for a given weekly alcohol intake, this excess incidence of cirrhosis is lower if the alcohol is consumed with meals and not every day.

## References

[1] Rehm J, Taylor B, Mohapatra S (2010). Alcohol as a risk factor for liver cirrhosis: a systematic review and meta-analysis. Drug Alcohol Rev.

[2] Corrao G, Bagnardi V, Zambon A, La Vecchia C (2004). A meta-analysis of alcohol consumption and the risk of 15 diseases. Prev Med.

[3] Yang L, Zhou M, Sherliker P (2012). Alcohol drinking and overall and cause-specific mortality in China: nationally representative prospective study of 220,000 men with 15 years of follow-up. Int J Epidemiol.

[4] Persson EC, Schwartz LM, Park Y (2013). Alcohol consumption, folate intake, hepatocellular carcinoma, and liver disease mortality. Cancer Epidemiol Biomarkers Prev.

[5] Goh GB, Chow WC, Wang R, Yuan JM, Koh WP (2014). Coffee, alcohol and other beverages in relation to cirrhosis mortality: the Singapore Chinese Health Study. Hepatology.

[6] Liu B, Balkwill A, Roddam A, Brown A, Beral V, Million Women Study Collaborators (2009). Separate and joint effects of alcohol and smoking on the risks of cirrhosis and gallbladder disease in middle-aged women. Am J Epidemiol.

[7] Askgaard G, Tolstrup JS (2015). Alcohol drinking frequency and risk of alcoholic cirrhosis in middle-aged women and men: results from a population-based cohort study. J Hepatol.

[8] Bellentani S, Saccoccio G, Costa G (1997). Drinking habits as cofactors of risk for alcohol induced liver damage. The Dionysos Study Group. Gut.

[9] Kamper-Jorgensen M, Gronbaek M, Tolstrup J, Becker U (2004). Alcohol and cirrhosis: dose–response or threshold effect?. J Hepatol.

[10] Becker U, Gronbaek M, Johansen D, Sorensen TI (2002). Lower risk for alcohol-induced cirrhosis in wine drinkers. Hepatology.

[11] Klatsky AL, Friedman GD, Armstrong MA, Kipp H (2003). Wine, liquor, beer, and mortality. Am J Epidemiol.

[12] Tuyns AJ, Pequignot G (1984). Greater risk of ascitic cirrhosis in females in relation to alcohol consumption. Int J Epidemiol.

[13] Green J, Reeves GK, Floud S (2018). Cohort profile: the Million Women Study. Int J Epidemiol.

[14] (2018). The Million Women Study. http://www.millionwomenstudy.org/.

[15] Liu B, Young H, Crowe FL (2011). Development and evaluation of the Oxford WebQ, a low-cost, web-based method for assessment of previous 24 h dietary intakes in large-scale prospective studies. Public Health Nutr.

[16] WHO (1992). International Statistical Classification of Diseases and Related Health Problems. Tenth Revision.

[17] Whitlock G, Clark T, Vander Hoorn S (2001). Random errors in the measurement of 10 cardiovascular risk factors. Eur J Epidemiol.

[18] Wood AM, Kaptoge S, Butterworth AS (2018). Risk thresholds for alcohol consumption: combined analysis of individual-participant data for 599 912 current drinkers in 83 prospective studies. Lancet.

[19] NHS Digital (2017). https://www.digital.nhs.uk/.

[20] Information Services Division (2017). http://www.isdscotland.org/.

[21] Plummer M (2004). Improved estimates of floating absolute risk. Stat Med.

[22] Townsend P, Phillimore P, Beattie A (1988). Health and deprivation: inequality and the north.

[23] NatCen Social Research and UCL (2017). Health survey for England 2016. Summary of key findings. https://files.digital.nhs.uk/pdf/s/q/hse2016-summary.pdf.

[24] Gentry RT (2000). Effect of food on the pharmacokinetics of alcohol consumption. Alcohol Clin Exp Res.

[25] Britton A, Ben-Shlomo Y, Benzeval M, Kuh D, Bell S (2015). Life course trajectories of alcohol consumption in the United Kingdom using longitudinal data from nine cohort studies. BMC Medicine.

