# Supplementary material for: Alcohol drinking patterns and liver cirrhosis risk: analysis of the prospective UK Million Women Study
Source: Lancet Public Health. 2018 Nov 22;4(1):e41–8. doi: 10.1016/S2468-2667(18)30230-5 (PMC6323353; doi:10.1016/S2468-2667(18)30230-5)
Supplement: Supplementary appendix [file mmc1.pdf]

THE LANCET
Public Health

Supplementary appendix

This appendix formed part of the original submission and has been peer reviewed. We post it as supplied by the authors.

Supplement to: Simpson RF, Hermon C, Liu B, et al. Alcohol drinking patterns and liver cirrhosis risk: analysis of the prospective UK Million Women Study. *Lancet Public Health* 2018; published online Nov 21. http://dx.doi.org/10.1016/S2468-2667(18)30230-5.

Alcohol drinking patterns and liver cirrhosis risk: analysis of the prospective UK Million Women Study

Supplementary Appendix

Rachel F. Simpson MB BCh,[1] Carol Hermon MSc,[1] Bette Liu DPhil,[2] Jane Green DPhil,[1] Gillian K. Reeves PhD[1], Valerie Beral FRS,[1] Sarah Floud PhD[1] for the Million Women Study Collaborators

[1] Cancer Epidemiology Unit, Nuffield Department of Population Health, University of Oxford, UK

[2]School of Public Health and Community Medicine, University of New South Wales, Sydney 2052, Australia

CONTENTS

Page 3: Membership of the Million Women Study Advisory Committee

Page 3: List of Million Women Study coordinating centre staff

Page 3: List of NHS Breast Cancer Screening Programme collaborating centres

Page 4-5: Remeasured alcohol consumption using data from the 24-hour recall questionnaire

Page 6-9: Supplementary Tables

Page 6: eTable1: Alcohol intake reported 11 years after baseline, by categories of intake reported at baseline

Page 7: eTable2: Relative risk of cirrhosis comparing alcohol consumption with meals to without meals, effect of adjusting for potential confounding factors

Page 8: eTable3: Relative risk of cirrhosis comparing alcohol consumption daily to less than daily, effect of adjusting for potential confounding factors (restricted to those reporting consumption of ≥7 drinks/week)

Page 9: eTable4: Baseline characteristics by type of alcohol consumed in all women reporting type

Page 10-11: Supplementary Figures

Page 10: eFigure1: Relative risk of liver cirrhosis by the amount of alcohol consumed and whether it was usually with meals, using information on alcohol consumption at recruitment for those with missing values at baseline.

Page 11: eFigure2: Relative risk of liver cirrhosis by amount of alcohol consumed and frequency of alcohol consumption, in women who consumed at least 7 drinks/week, using information on alcohol consumption at recruitment for those with missing values at baseline.

Membership of the Million Women Study Advisory Committee
Emily Banks, Valerie Beral, Lucy Carpenter, Carol Dezateux, Jane Green, Julietta Patnick, Richard Peto, Cathie Sudlow.

List of Million Women Study coordinating centre staff
Simon Abbott, Rupert Alison, Krys Baker, Angela Balkwill, Isobel Barnes, Valerie Beral, Judith Black, Roger Blanks, Anna Brown, Benjamin Cairns, Andrew Chadwick, Dave Ewart, Sarah Floud, Kezia Gaitskell, Toral Gathani, Laura Gerrard, Adrian Goodill, Jane Green, Lynden Guiver, Carol Hermon, Darren Hogg, Isobel Lingard, Sau Wan Kan, Nicky Langston, Kirstin Pirie, Alison Price, Gillian Reeves, Keith Shaw, Emma Sherman, Rachel Simpson, Helena Strange, Siân Sweetland, Ruth Travis, Lyndsey Trickett, Anthony Webster, Clare Wotton, Lucy Wright, Owen Yang, Heather Young.

List of NHS Breast Cancer Screening Programme collaborating centres
The following NHS Breast Screening Centres took part in the recruitment and breast screening follow-up for the Million Women Study: Avon, Aylesbury, Barnsley, Basingstoke, Bedfordshire and Hertfordshire, Cambridge and Huntingdon, Chelmsford and Colchester, Chester, Cornwall, Crewe, Cumbria, Doncaster, Dorset, East Berkshire, East Cheshire, East Devon, East of Scotland, East Suffolk, East Sussex, Gateshead, Gloucestershire, Great Yarmouth, Hereford and Worcester, Kent, Kings Lynn, Leicestershire, Liverpool, Manchester, Milton Keynes, Newcastle, North Birmingham, North East Scotland, North Lancashire, North Middlesex, North Nottingham, North of Scotland, North Tees, North Yorkshire, Nottingham, Oxford, Portsmouth, Rotherham, Sheffield, Shropshire, Somerset, South Birmingham, South East Scotland, South East Staffordshire, South Derbyshire, South Essex, South Lancashire, South West Scotland, Surrey, Warrington Halton St Helens and Knowsley, Warwickshire Solihull and Coventry, West Berkshire, West Devon, West London, West Suffolk, West Sussex, Wiltshire, Winchester, Wirral, Wycombe.

Remeasured alcohol consumption using data from the 24-hour recall questionnaire

The importance of using remeasurements of alcohol consumption
It is important to remeasure alcohol consumption to assess whether the intakes reported at baseline have changed over time, since alcohol consumption can vary over a person's lifetime.[1] Changes in consumption need to be taken into account as do measurement error and regression dilution bias when describing the relationship between various levels of alcohol intake and the risk of cirrhosis.[2]

The 24-hour recall questionnaire
The on-line 24-hour recall questionnaire, asking about diet and alcohol consumption on the previous day, was completed on average 11 (SD 2) years after baseline by 19,293 women from the 401,806 baseline population of drinkers. The on-line questionnaires were completed between 30th June 2010 and 31st March 2017.

Some women completed more than one 24-hr recall questionnaire, but we used the first questionnaire completed by each woman to prevent any bias created by using multiple questionnaires filled out by a particular participant. To be invited to complete a 24hr recall, women had to have a valid e-mail address. The 24-hour recall questionnaires were sent to participants on a randomly selected day of the week and responses that were not provided within 48 hours were disregarded.

In the 24-hour recall, women were asked whether they had consumed any alcohol on the previous day. This question had to be answered to proceed with the questionnaire, so there were no missing data here. If the participant answered that she had drunk alcohol, she had to tick at least one amount for an alcoholic beverage type before continuing to the next page of the questionnaire. However, women did not have to record their intake of all beverage types and so if no quantity was specified, we regarded intake of that beverage type as zero. Women were asked to report separately their consumption of five different alcoholic beverage groups: wine in wine glasses; beer, lager, or cider in pints; sherry, fortified wine, or port in sherry or port glasses; spirits in measures; and other alcoholic drinks in units. For the specified measures for each beverage type, women were given the option of answering that they had consumed none, ½, 1, 2, 3, 4, 5, or 6+ servings on the previous day. We took 6+ as being 6 for the purposes of calculations. If any wine consumption was recorded, the participant was asked to specify the glass size as small (125ml), medium (175ml), or large (250ml). If a participant ticked more than one glass size, the midpoint value was taken, for example 150ml if boxes relating to both small and medium glasses were ticked. If a participant had recorded drinking wine but had not specified the size of the glass, this was taken as 175ml (a medium glass) since this was the modal group for glass size.

We calculated the alcohol consumed from the 24-hour recall in grams by adding up the amounts of the different types of alcohol. We took a small glass of wine as 12 grams of alcohol, presupposing an alcohol content of 12%, a pint of beer, lager, or cider as 16 grams, assuming an alcohol content of 3·5%, and a port glass, spirits shot, or unit of another alcoholic drink as 8 grams.[3]

Allowing for changes in consumption over time and measurement error in these analyses
We used the number of drinks and drinking habits reported on the baseline questionnaire, completed in median year 2001 (3 years after recruitment) to define the categories for drinks/week, mealtime habits, and weekly frequency of consumption. The recruitment questionnaire was used to define the type of alcohol consumed (as questions on the type of alcohol were not repeated 3 years after recruitment). To account for changes in alcohol consumption pattern, regression dilution bias, and to minimise measurement error,[2] we assigned each baseline category an average value in grams/week using data from the on-line 24-hour recall questionnaire.

We calculated the average amount of alcohol consumed in grams/week by women in each category at baseline by summing the relevant mean amounts for each day of the week. We calculated means by day of the week as alcohol intake was higher at the weekend and we wanted to account for this as well as slightly different numbers of responses for each day of the week. The standard deviations presented for the average weekly consumptions from the 24-hour recall were calculated as the square root of the sum of the variance of alcohol intake for each day of the week for each category of consumption.

References

1. Britton A, Ben-Shlomo Y, Benzeval M, Kuh D, Bell S. Life course trajectories of alcohol consumption in the United Kingdom using longitudinal data from nine cohort studies. *BMC Medicine 2015*; **13**: 47. DOI: 10.1186/s12916-015-0273-z
2. Whitlock G, Clark T, Vander Hoorn S et al. Random errors in the measurement of 10 cardiovascular risk factors. Eur J Epidemiol 2001; **17**: 907-909.
3. Brown L. Health survey for England 2015. Adult alcohol consumption [online]. Leeds: Health and Social Care Information Centre; 2016. Available from: http://www.content.digital.nhs.uk/catalogue/PUB22610/HSE2015-Adult-alc.pdf

eTable1: Alcohol intake reported 11 years after baseline, by categories of intake reported at baseline.

	**Average number of drinks per week reported 11 years after baseline (sd)**			
	**1-2 drinks/week at baseline**	**3-6 drinks/week at baseline**	**7-14 drinks/week at baseline**	**15+ drinks/week at baseline**
**All women**	**2·5 (1.9)**	**5·2 (2·7)**	**10·0 (3·7)**	**18·0 (5·2)**
Usually drink with meals (at baseline)	2·6 (1·9)	5·2 (2·7)	10·1 (3·5)	18·0 (5·0)
Usually do not drink with meals or varies (at baseline)	2·2 (1·9)	5·1 (2·8)	10·0 (3·9)	18·0 (5·4)
Less than daily consumption (at baseline)			9.9 (3.8)	15.6 (5.0)
Daily consumption (at baseline)			10.3 (3.5)	19.7 (5.2)

eTable2: Relative risk of cirrhosis comparing alcohol consumption with meals to without meals, effect of adjusting for potential confounding factors*

	**Relative risk (95% CI) in alcohol consumption with vs without meals**	**Chi-squared value**	**% attenuation in χ^2**
**Stratified by year of birth, year of recruitment and adjusted for region**	0·52 (0·47-0·58)	152	
**As above, with additional adjustment for alcohol**			
Alcohol amount	**0·57 (0·51-0·63)**	**117**	**24%**
**As above, with additional adjustment for amount of alcohol and each of the following factors, one at a time**	**0·57 (0·51-0·63)**	**117**	
Smoking	0·65 (0·58-0·72)	66	43%
Body mass index	0·59 (0·53-0·66)	98	16%
Deprivation quintile	0·59 (0·53-0·65)	99	15%
Oral contraceptive pill use	0·57 (0·51-0·63)	116	0%
Use of menopausal hormones	0·57 (0·51-0·63)	115	1%
Additional adjustment by all the above factors	**0·69 (0·62-0·77)**	**47**	**59%**

*Adjustment for confounders includes all participants, including those with unknown values

eTable3: Relative risk of cirrhosis comparing alcohol consumption daily to less than daily, effect of adjusting for potential confounding factors (restricted to those reporting consumption of $\geq$7 drinks/week)

	**Relative risk (95% CI) in alcohol consumption daily vs <daily**	**Chi-squared value**	**% attenuation in χ^2**
**Stratified by year of birth, year of recruitment and adjusted for region**	1·75 (1·53-2·00)	67	
**As above, with additional adjustment for variables related to alcohol consumption, one at a time:**			
Alcohol amount	1·47 (1·28-1·68)	30	56%
Meals	1·91 (1·67-2·19)	88	-33%
Type of alcohol	1·78 (1·56-2·04)	71	-7%
**Additional adjustment by all the above factors**	**1·61 (1·40-1·86)**	**45**	**32%**
**As above, with additional adjustment for alcohol related variables and each of the following variables, one at a time:**	**1·61 (1·40-1·86)**	**45**	
Smoking	1·55 (1·35-1·78)	38	16%
Body mass index	1·64 (1·43-1·89)	49	-7%
Deprivation quintile	1·64 (1·43-1·89)	49	-8%
Oral contraceptive pill use	1·61 (1·40-1·86)	45	0%
Use of menopausal hormones	1·62 (1·40-1·86)	46	-1%
**Additional adjustment by all the above factors**	**1·61 (1·40-1·85)**	**44**	**3%**

eTable4: Baseline characteristics by type of alcohol consumed in all women reporting type (n=393,900)

	Alcohol type			
	**Wine only** n=135,628	**Spirits only** n=22,020	**Lager/beer/cider only** n=10,486	**Mixed** n=225,766
**Alcohol consumption at baseline**				
g/week, mean (SD)	84 (68)	87 (70)	92 (78)	93 (71)
Usually drink with meals, % (n)	64 (87,360)	14 (3,022)	14 (1492)	48 (108,372)
**Alcohol consumption 11 years later**				
g/week, mean (SD)	100 (47)	70 (42)	75 (40)	105 (47)
**Characteristics at baseline**				
Age, mean (sd)	59 (5)	60 (5)	58 (4)	59 (5)
BMI (kg/m^2), mean (sd)	25 (4)	26 (5)	26 (4)	26 (4)
Most deprived quintile*, % (n)	10 (13,703)	24 (5,281)	36 (3,769)	11 (25,078)
Current smoker, % (n)	9 (11,834)	26 (5,597)	31 (3,156)	9 (20,536)
Current use of hormone therapy, % (n)	30 (39,954)	28 (6,042)	29 (2,929)	31 (69,244)
**Follow-up**				
Person-years (1000s)	1974057	311698	149575	3287364
Years follow-up per woman	14·6	14·2	14·3	14·6
New cases of cirrhosis	390	186	71	859

*From recruitment

Percentages are calculated for women who have data on type of alcohol and with data for variables of interest (those with missing values are excluded)

eFigure 1. Relative risk of liver cirrhosis by the amount of alcohol consumed and whether it was usually with meals, using information on alcohol consumption at recruitment for those with missing values at baseline

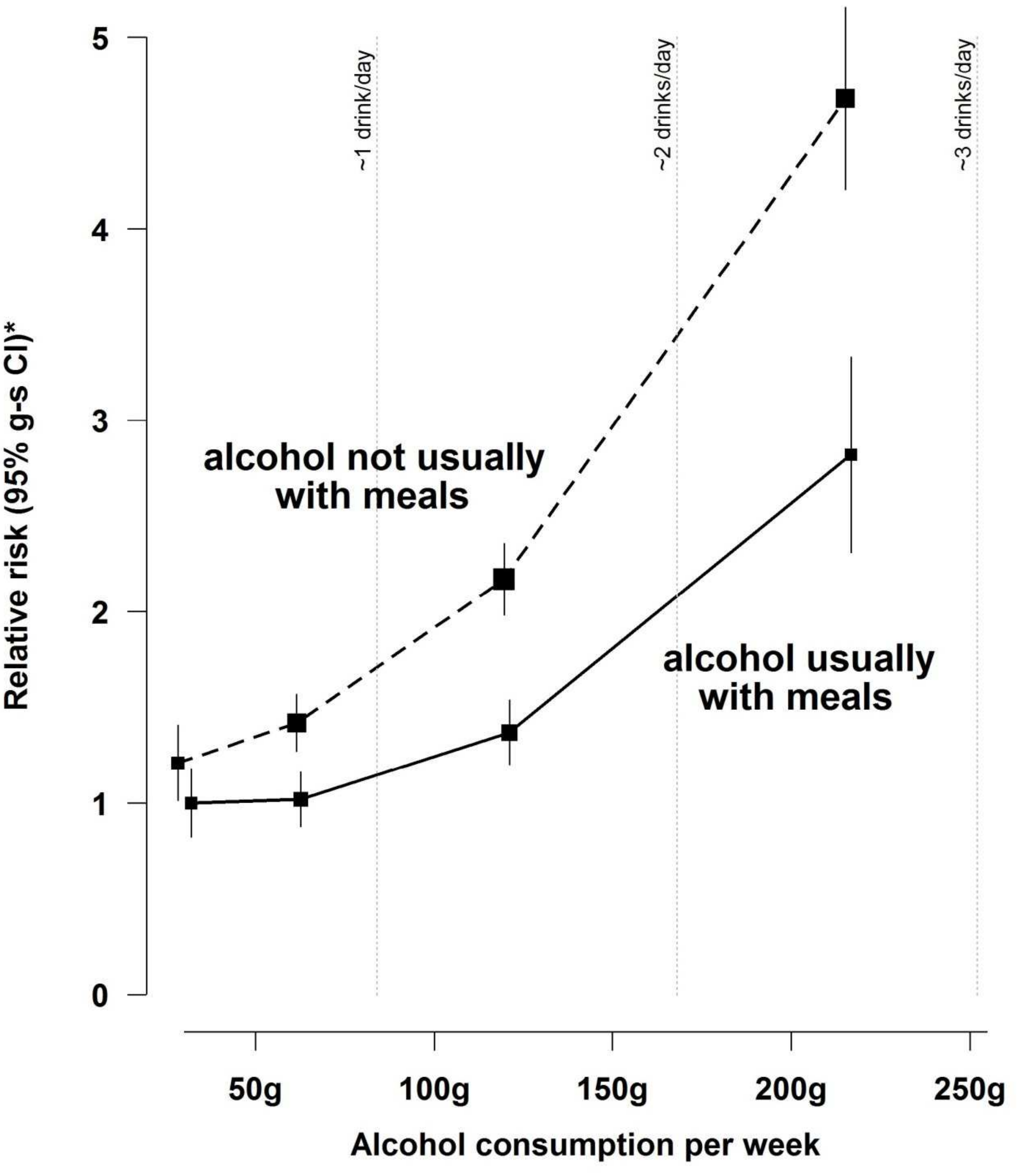

*Relative risk and 95% g-s confidence intervals (CI) for liver cirrhosis by amount of alcohol consumed compared to consumption of 1-2 drinks (mean 32g) per week with meals (relative risk=1.0), adjusted for region, body mass index, deprivation quintile, smoking, use of oral contraceptives and menopausal hormones and stratified by year of birth and year completed baseline questionnaire.
The relative risks are for categories of 1-2, 3-6, 7-14 ,15+ drinks/week plotted against the remeasured averages in each category (with meals 32, 63, 121, 217 grams/week respectively; without meals 28, 62, 120, 215 grams/week respectively).

eFigure 2. Relative risk of liver cirrhosis by amount of alcohol consumed and frequency of alcohol consumption, in women who consumed at least 7 drinks/week, using information on alcohol consumption at recruitment for those with missing values at baseline

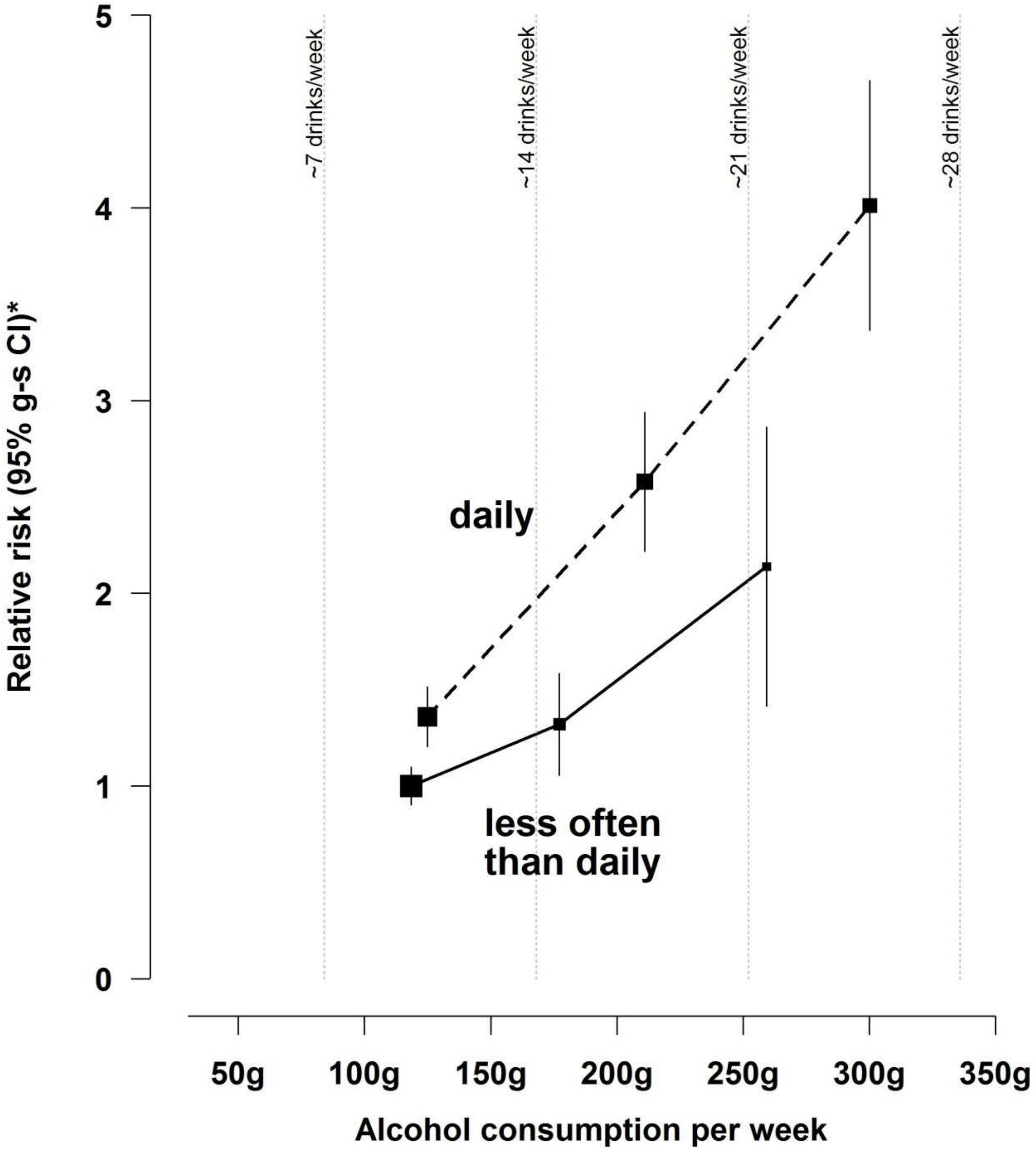

*Relative risk and 95% g-s confidence intervals (CI) for liver cirrhosis by amount of alcohol consumed compared to consumption of 7-14 drinks (mean 118g) per week less often than daily (relative risk=1.0), adjusted for region, body mass index, deprivation quintile, smoking, use of oral contraceptives, menopausal hormones, meal time habits and type of alcohol and stratified by year of birth and year completed baseline questionnaire.
The relative risks are for categories of 7-14 ,15-21, 22+ drinks/week plotted against the remeasured averages in each category (less often than daily 118, 177, 259 grams/week respectively; daily 125, 211, 300 grams/week respectively).
